# Explainable Artificial Intelligence in Radiological Cardiovascular Imaging—A Systematic Review

**DOI:** 10.3390/diagnostics15111399

**Published:** 2025-05-31

**Authors:** Matteo Haupt, Martin H. Maurer, Rohit Philip Thomas

**Affiliations:** Department of Diagnostic and Interventional Radiology, Carl von Ossietzky Universität Oldenburg, 26129 Oldenburg, Germany

**Keywords:** explainable artificial intelligence, cardiovascular imaging, deep learning, model interpretability, Grad-CAM, cardiac CT, cardiac MRI, echocardiography

## Abstract

**Background:** Artificial intelligence (AI) and deep learning are increasingly applied in cardiovascular imaging. However, the “black box” nature of these models raises challenges for clinical trust and integration. Explainable Artificial Intelligence (XAI) seeks to address these concerns by providing insights into model decision-making. This systematic review synthesizes current research on the use of XAI methods in radiological cardiovascular imaging. **Methods:** A systematic literature search was conducted in PubMed, Scopus, and Web of Science to identify peer-reviewed original research articles published between January 2015 and March 2025. Studies were included if they applied XAI techniques—such as Gradient-Weighted Class Activation Mapping (Grad-CAM), Shapley Additive Explanations (SHAPs), Local Interpretable Model-Agnostic Explanations (LIMEs), or saliency maps—to cardiovascular imaging modalities, including cardiac computed tomography (CT), magnetic resonance imaging (MRI), echocardiography and other ultrasound examinations, and chest X-ray (CXR). Studies focusing on nuclear medicine, structured/tabular data without imaging, or lacking concrete explainability features were excluded. Screening and data extraction followed PRISMA guidelines. **Results:** A total of 28 studies met the inclusion criteria. Ultrasound examinations (*n* = 9) and CT (*n* = 9) were the most common imaging modalities, followed by MRI (*n* = 6) and chest X-rays (*n* = 4). Clinical applications included disease classification (e.g., coronary artery disease and valvular heart disease) and the detection of myocardial or congenital abnormalities. Grad-CAM was the most frequently employed XAI method, followed by SHAP. Most studies used saliency-based techniques to generate visual explanations of model predictions. **Conclusions:** XAI holds considerable promise for improving the transparency and clinical acceptance of deep learning models in cardiovascular imaging. However, the evaluation of XAI methods remains largely qualitative, and standardization is lacking. Future research should focus on the robust, quantitative assessment of explainability, prospective clinical validation, and the development of more advanced XAI techniques beyond saliency-based methods. Strengthening the interpretability of AI models will be crucial to ensuring their safe, ethical, and effective integration into cardiovascular care.

## 1. Introduction

Cardiovascular diseases remain the leading cause of mortality worldwide and impose a substantial healthcare burden [1]. Imaging modalities such as ultrasound, cardiac magnetic resonance imaging (MRI), cardiac computed tomography (CT), and X-rays are pivotal to cardiovascular diseases’ diagnosis and management, providing detailed visualizations of cardiac structures and functions. In recent years, advanced artificial intelligence (AI) techniques—particularly deep learning—have shown promising results in analyzing these cardiovascular images [2]. AI systems can assist in various imaging-based tasks, including disease classification, the segmentation of anatomical structures, and even image reconstruction [3]. By leveraging large datasets of cardiac images and related clinical data, such as electrocardiograms and health records, deep learning models have achieved a high accuracy in identifying subtle imaging patterns and predicting patient outcomes [4]. This convergence of AI with radiology and cardiology holds great potential to enhance diagnostic precision, reduce workflow burdens, and enable more personalized cardiac care.

Despite these advancements, a critical challenge has emerged: many state-of-the-art AI models function as “black boxes” in the clinical context [5]. Deep neural networks are powerful, but their predictions are often not transparent, which makes clinicians less willing to trust an automated diagnosis or risk estimate if they cannot understand how it was made [6]. A lack of interpretability and transparency not only undermines user confidence but also conflicts with the principles of evidence-based medicine and accountability in patient care [7]. For example, if an AI model flags a cardiac MRI as showing early signs of heart failure, the cardiologist needs to know why—which image features or regions influenced that prediction—before acting on it. Regulatory and ethical considerations further drive this need; guidelines and frameworks increasingly call for AI decisions in healthcare to be explainable to ensure fairness, safety, and informed consent [8]. In response to these concerns, the field of explainable AI (XAI) has risen to prominence as a means to “open the black box” and make AI’s decision-making process more interpretable.

XAI encompasses a variety of methods designed to elucidate how AI models arrive at their outputs. In the context of deep learning for medical imaging, XAI techniques often provide post hoc explanations by highlighting important features in the input image or by offering interpretable representations of the model’s internal reasoning. Because medical imaging is a predominantly visual domain, many explainability approaches rely on saliency maps and related visualization tools [3]. A prominent example is Gradient-Weighted Class Activation Mapping (Grad-CAM), which produces class-discriminative heatmaps by using the gradients of a target concept (e.g., disease class) flowing into the final convolutional layer [9]. Grad-CAM computes the importance weights of each feature map through the global average pooling of the gradients and combines them into a heatmap that highlights the most influential regions in the input image. It is particularly effective for convolutional neural networks in image classification and is widely used due to its simplicity and visual interpretability.

Grad-CAM belongs to the broader family of saliency-based methods, which visualize the influence of input features on the model output. However, unlike classical saliency maps—which calculate the gradient of the output with respect to individual input pixels—Grad-CAM operates on higher-level feature maps and yields more structured, semantically meaningful explanations. While classical saliency maps can be noisy and sensitive to pixel-level perturbations, several refinements have been proposed to enhance their interpretability. SmoothGrad, for instance, averages multiple saliency maps generated from noisy input variants to reduce visual noise, while LayerCAM improves spatial resolution by weighting activations directly, rather than relying solely on gradients [10]. These enhanced methods offer more stable and anatomically meaningful outputs in complex imaging scenarios.

Other common XAI methods include Shapley Additive Explanations (SHAPs) and Local Interpretable Model-Agnostic Explanations (LIMEs), which provide feature-level insights through fundamentally different mechanisms [11]. The SHAP is grounded in cooperative game theory and estimates the contribution of each input feature by approximating Shapley values. In imaging, SHAPs are often applied to radiomics features or deep feature embeddings, and although it provides theoretically sound attributions, it is computationally intensive. The LIME explains individual predictions by learning a simple, interpretable surrogate model (e.g., linear regression) around a given input, based on perturbed samples. In image domains, this involves dividing the input into superpixels and analyzing how masking or altering each region affects the prediction. While the LIME is model-agnostic and intuitive, it can suffer from instability depending on the perturbation strategy and segmentation granularity [11].

**Figure 1:**
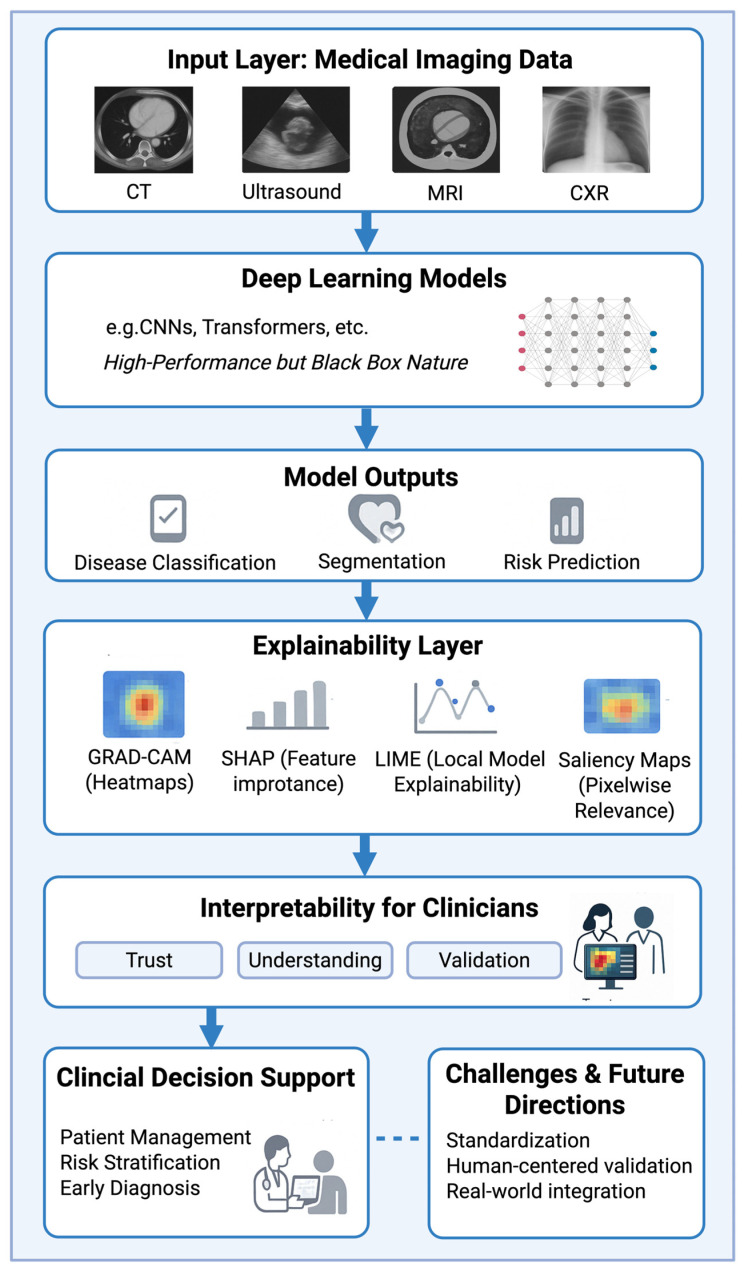
Workflow from medical imaging to clinical decision support using explainable artificial intelligence (XAI).

These techniques, originally developed for general machine learning, have been adapted to imaging by partitioning images into segments or using features learned by the network. Beyond visual explanations, there are also emerging approaches such as case-based reasoning, where the model retrieves similar past cases to justify a prediction, and textual explanations, where the model generates human-readable descriptions of its findings [3]. Together, these XAI tools aim to bridge the gap between complex AI algorithms and clinical interpretability. A conceptual overview of the integration of deep learning models, explainability methods, and clinical decision-making in cardiovascular imaging is illustrated in Figure 1.

The clinical relevance of XAI in cardiovascular imaging is significant: by revealing how an AI model arrives at its conclusions, XAI allows clinicians to compare the AI’s reasoning with their own medical expertise [2]. This not only builds trust in the technology but can also uncover new imaging biomarkers or patterns that clinicians might not have been explicitly aware of. Indeed, initial studies have shown that when deep learning models are accompanied by explanations, physicians are more willing to consider the AI’s suggestion and can better identify cases where the model might have erred [2]. Explainability also facilitates the troubleshooting of AI models: if the highlighted region or feature does not make sense, such as when the model focuses on an artifact or unrelated anatomy, developers can recognize that it may be relying on spurious correlations and make adjustments. Ultimately, XAI methods serve as a crucial step towards integrating AI into routine cardiac imaging workflows, where interpretability is essential for medicolegal responsibility and clinician acceptance [7]. 

This systematic review aims to provide an overview of how Explainable Artificial Intelligence has been applied across different cardiovascular imaging modalities, to describe the XAI techniques and clinical tasks addressed, and to outline current strengths and limitations. By summarizing the available studies, we seek to present the state of the research, identify methodological trends and gaps, and offer perspectives for future developments toward clinically meaningful and robust applications of explainable AI in cardiovascular imaging.

Medical imaging data from cardiac computed tomography (CT), magnetic resonance imaging (MRI), echocardiography, and chest X-rays (CXRs) are processed using deep learning models, such as convolutional neural networks (CNNs) or transformer architectures, which are characterized by their high performance but black box nature. The resulting model outputs, including disease classification, the segmentation of anatomical structures, and risk prediction, are subsequently interpreted using explainability methods such as Gradient-Weighted Class Activation Mapping (Grad-CAM), Shapley Additive Explanations (SHAPs), Local Interpretable Model-Agnostic Explanations (LIMEs), and pixelwise saliency maps. These explainability techniques enhance clinical decision-making by improving trust, understanding, and clinical validation among clinicians. Finally, XAI supports patient management, risk stratification, and early diagnosis. Challenges and future directions include the need for standardization, human-centered validation, and real-world clinical integration.

## 2. Methods

### 2.1. Literature Search Strategy

A systematic literature search was conducted across three major databases—PubMed, Scopus, and Web of Science—to identify original research articles applying XAI methods in the context of cardiovascular imaging. The search covered the period from January 2015 to March 2025 and was limited to studies published in English. The search strategy was designed to combine terms relating to cardiovascular imaging modalities (e.g., cardiac MRI, coronary CT angiography, and echocardiography), artificial intelligence techniques (e.g., deep learning, convolutional neural networks), and XAI approaches (e.g., Grad-CAM, SHAP, LIME, saliency maps, layer-wise relevance propagation, among others). To ensure a clear focus on explainability, at least one XAI-related term was required to appear in the title or abstract of each study.

The search queries were tailored to the syntax and indexing of each database and are documented in detail in the Supplementary Material (Table S1). In PubMed, a highly structured Boolean search was employed to cover cardiovascular anatomy, imaging modalities, AI techniques, and explainability keywords, excluding reviews. In Scopus, the query targeted titles and abstracts containing XAI terms, cardiovascular imaging modalities, and general imaging keywords. The Web of Science strategy included terms related to XAI in the title or abstract and required mention of both cardiovascular imaging and AI techniques, restricted to non-review articles published in English between 2015 and March 2025.

### 2.2. Study Selection

After automated removal of duplicates, 146 unique records were screened. In a two-step process, titles and abstracts were first reviewed for relevance, followed by a full-text assessment of 63 potentially eligible studies based on predefined inclusion and exclusion criteria.

Studies were included if they applied explainable AI techniques to cardiovascular imaging data, with a focus on diagnostic, classification, or risk prediction tasks. Only original research articles were considered, and the analyzed data had to be image-based, such as CT, MRI, echocardiography, or other ultrasound examinations. Studies were excluded if they did not analyze imaging data, did not focus on cardiovascular imaging, or lacked a concrete and reproducible XAI technique. Crucially, studies relying on nuclear medicine techniques—such as positron emission tomography or single-photon emission computed tomography—were excluded due to their fundamentally different acquisition principles and clinical workflows. In addition, articles that applied explainable AI only to structured or tabular clinical data, without reference to imaging data, were not considered. Editorials, reviews, preprints, and conference abstracts were also excluded.

**Figure 2:**
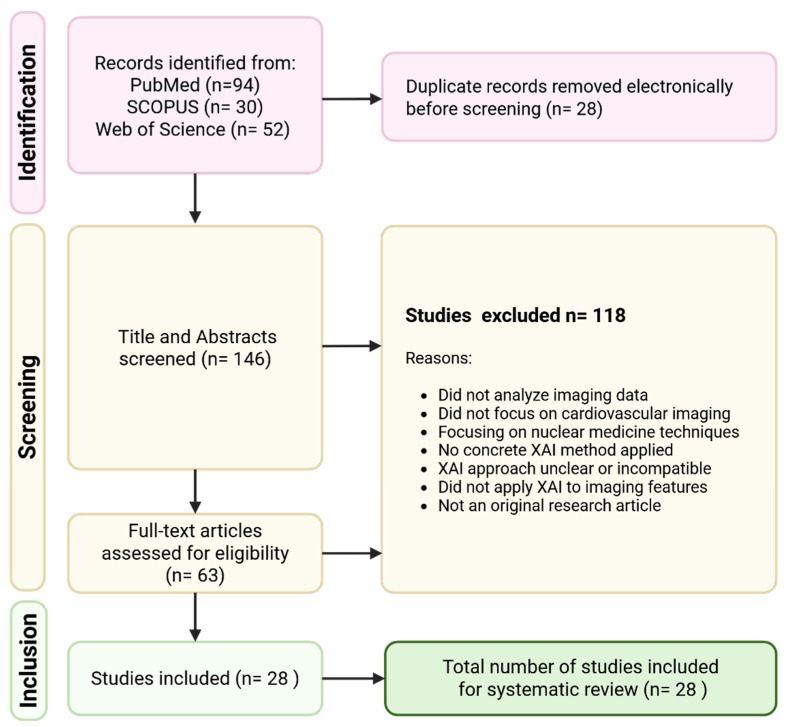
Preferred Reporting Items for Systematic Reviews and Meta-Analyses (PRISMA) flowchart of the study selection process: A total of 176 records were identified through database searches (PubMed, SCOPUS, and Web of Science). After removing 28 duplicates, 146 titles and abstracts were screened. Following the exclusion of 118 studies based on predefined criteria, 63 full-text articles were assessed for eligibility. Ultimately, 28 studies were included in the systematic review.

Ultimately, 28 studies met all inclusion criteria and were incorporated into the final synthesis. The full selection process is visualized in Figure 2 as a Preferred Reporting Items for Systematic Reviews and Meta-Analyses (PRISMA) Flow Diagram. The included studies were synthesized narratively and grouped by imaging modality to facilitate structured comparison of XAI methods and applications.

## 3. Results

### 3.1. Overview of Included Studies

A total of 28 studies met the predefined inclusion criteria and were included in the final synthesis. These studies span a variety of radiological imaging modalities used in cardiovascular diagnostics, including CT, MRI, echocardiography and other ultrasound examinations, and chest radiography (CXR). The studies were grouped and analyzed according to the imaging modality to facilitate a structured comparison.

Across the dataset, a broad range of XAI techniques were employed. The most frequently used methods were Grad-CAM, applied in 15 of the 28 studies, followed by SHAP, which was used in 9 studies. Other reported methods included LIMEs, saliency maps (including SmoothGrad and LayerCAM), and Class Activation Maps (CAMs). The frequent application of Grad-CAM reflects its wide adoption in convolutional neural network (CNN)-based image classification tasks, likely due to its intuitive visual feedback on model decision-making.

The following sections provide a detailed breakdown of the included studies, structured by imaging modality.

### 3.2. Computed Tomography

Nine of the included studies applied Explainable Artificial Intelligence techniques in the context of CT-based cardiovascular imaging (Table 1). The majority of these focused on contrast-enhanced coronary CT angiography (CCTA), which remains a cornerstone in the non-invasive imaging of coronary artery disease. Two studies additionally evaluated non-coronary applications, including abdominal and chest CT.

Several works aimed to improve disease detection and classification from CCTA. Gerbasi et al. proposed a visually explainable deep learning pipeline using a Multi-Axis Vision Transformer for automated CAD-RADS scoring, integrating SHAPs to enhance interpretability [12]. Similarly, Penso et al. employed a ConvMixer-based token-mixer architecture for CAD-RADS classifications on multiplanar reconstruction images, complemented by Grad-CAM-based visualizations [13]. 

Other studies focused on prediction tasks in CT angiography. Lopes et al. developed a machine learning model to identify insufficient contrast enhancement in coronary CTA, highlighting the role of SHAPs in interpreting the impact of test bolus parameters and patient features [14]. In a related vascular application, Sakai et al. classified culprit calcified carotid plaques in embolic strokes of undetermined sources using SHAPs to explain decisions based on neck CT angiography [15]. Next, Candemir et al. developed a fully automated deep learning system for the detection and weakly supervised localization of coronary artery atherosclerosis on CCTA scans [16]. A 3D convolutional neural network (3D-CNN) was used to classify vessels as healthy or diseased and to highlight discriminative regions using Grad-CAM. Achieving a high negative predictive value (NPV) of 96.1%, the study demonstrates the potential of AI-based systems to assist physicians in efficiently ruling out coronary atherosclerosis in patients with acute chest pain.

Pathology-specific models were also presented. Wang et al. developed SOSPCNN, a Grad-CAM-enhanced convolutional network, to detect the Tetralogy of Fallot in contrast-enhanced cardiac CT [17]. Lo Iacono et al. proposed a SHAP-based radiomics pipeline to distinguish cardiac amyloidosis from aortic stenosis in contrast-enhanced CT, suggesting textural features as relevant discriminators [18].

Beyond contrast-enhanced protocols, Huo et al. introduced a weakly supervised 3D Grad-CAM framework to detect coronary artery calcium on non-contrast chest CT [19]. Additionally, Fu et al. applied SHAPs to radiomic features extracted from non-contrast abdominal CT to predict outcomes after renal angioplasty, extending XAI applications beyond a cardiac focus [20].

Together, these studies demonstrate the versatility of XAI across CT-based modalities, with Grad-CAM and SHAPs being the most commonly employed techniques to visualize decision-relevant image regions or rank feature importance.

**Table 1:** Overview of studies applying explainable AI (XAI) methods to cardiovascular and vascular CT imaging. Abbreviations: XAI: Explainable Artificial Intelligence; SHAPs: Shapley Additive Explanations; Grad-CAM: Gradient-Weighted Class Activation Mapping; CT: computed tomography; CCTA: Coronary Computed Tomography Angiography; CAD-RADS: Coronary Artery Disease—Reporting and Data System; ML: machine learning; MPR: multiplanar reconstruction.

Author	Year	XAI Method(s)	Imaging Modality	Aim of the Study	Ref.
Gerbasi et al.	2024	Deep SHAP	CCTA	To develop a fully automated, visually explainable deep learning pipeline using a Multi-Axis Vision Transformer for CAD-RADS scoring of CCTA scans, aiming to classify patients based on the need for further investigations and severity of coronary stenosis.	[12]
Sakai et al.	2024	SHAP	contrast-enhanced CT angiography	To classify culprit versus non-culprit calcified carotid plaques in embolic stroke of undetermined source using an explainable ML model with clinically interpretable imaging features.	[15]
Fu et al.	2024	SHAP	non-contrast abdominal CT	To predict early outcomes after percutaneous transluminal renal angioplasty in patients with severe atherosclerotic renal artery stenosis using CT-based radiomics.	[20]
Lo Iacono et al.	2023	SHAP	contrast-enhanced cardiac CT	To differentiate cardiac amyloidosis from aortic stenosis using radiomic features and machine learning.	[18]
Penso et al.	2023	Grad-CAM	CCTA	To classify coronary stenosis from MPR images using a ConvMixer-based token-mixer architecture according to CAD-RADS scoring.	[13]
Lopes et al.	2022	SHAP	CCTA	To use machine learning models to predict insufficient contrast enhancement in coronary CT angiography and interpret predictive features using SHAP.	[14]
Candemir et al.	2020	Grad-CAM	CCTA	Automated detection and weakly supervised localization of coronary artery atherosclerosis using a 3D-CNN.	[16]
Wang et al.	2021	Grad-CAM	Contrast-enhanced Cardiac CT	To develop an explainable AI model for recognizing Tetralogy of Fallot in cardiovascular CT images.	[17]
Huo et al.	2019	Grad-CAM	non-contrast chest CT	To detect coronary artery calcium using 3D attention-based deep learning with weakly supervised learning and to visualize predictions using 3D Grad-CAM.	[19]

### 3.3. Magnetic Resonance Imaging

Six studies in the final synthesis applied explainable AI methods to cardiovascular tasks based on MRI, with a focus on cardiac MRI and vascular-related imaging (Table 2). These studies addressed both structural and functional aspects of cardiovascular pathology and incorporated a diverse array of XAI techniques.

A central application was the assessment of myocardial tissue characteristics. Paciorek et al. developed deep learning models based on DenseNet-161 to classify cardiac pathology using T1 mapping and phase-sensitive inversion recovery sequences [21]. Grad-CAM visualizations were used to verify that the model attended to pathologically relevant myocardial regions, supporting clinical interpretability.

Zhang et al. utilized both cardiac MRI and angiography-derived data to predict microvascular obstructions in patients with STEMI undergoing a primary percutaneous coronary intervention [22]. The integration of SHAPs allowed for detailed insights into the role of angio-based microvascular resistance and other clinical parameters in model predictions.

Expanding beyond cardiac applications, Mouches et al. explored biological brain age predictions using multimodal MRI and a more specific T1-weighted and time-of-flight MR angiography [23]. Their use of SmoothGrad-based saliency maps enabled the identification of vascular and cortical structures most predictive of aging effects, providing a valuable model for cerebrovascular aging studies.

Cai et al. also linked perfusion-related MRI data with ultrasound-based carotid flows to model the cerebral perfusion status. SHAPs were employed to interpret the influence of physiological variables and imaging-derived metrics within the prediction model [24].

Beyond achieving a high diagnostic accuracy, Wang et al. also addressed the crucial aspect of model interpretability by applying XAI techniques [25]. Using guided Grad-CAM and Shapley value analyses, they visualized which cardiac regions and imaging modalities most influenced the AI’s decisions for specific cardiovascular diseases. This approach not only enhances clinical trust in AI-assisted diagnostics but also provides new insights into disease-specific imaging features that might otherwise remain underrecognized.

Lastly, the study by Sufian et al. addressed a broader systemic concern: algorithmic bias [26]. Utilizing data from both CT and MRI, the authors integrated SHAPs, LIMEs, and fairness-aware tools (e.g., Fairlearn) to detect and mitigate demographic bias in cardiovascular predictions. Their model represents an important contribution to the intersection of explainability, fairness, and generalizability in AI.

Taken together, these MRI-focused studies highlight both the diagnostic versatility of AI in soft tissue and vascular imaging and the potential of XAI techniques like SHAPs and Grad-CAM to ensure model transparency across a range of clinical tasks.

**Table 2:** Overview of studies applying explainable AI (XAI) methods to cardiovascular MRI. Abbreviations: XAI: Explainable Artificial Intelligence; SHAP: Shapley Additive Explanations; LIME: Local Interpretable Model-Agnostic Explanations; Grad-CAM: Gradient-Weighted Class Activation Mapping; MRI: magnetic resonance imaging; PPCI: primary percutaneous coronary intervention; STEMI: ST-Elevation Myocardial Infarction; PSIR: phase-sensitive inversion recovery.

Author	Year	XAI Method(s)	Imaging Modality	Aim of the Study	Ref.
Zhang et al.	2025	SHAP	Cardiac MRI	To predict microvascular obstruction using a machine learning model based on angio-based microvascular resistance and clinical data during PPCI in STEMI patients and to interpret the model using SHAP.	[22]
Wang et al.	2024	Grad-CAM, SHAP	Cardiac MRI	Development and evaluation of AI models for screening and diagnosis of multiple cardiovascular diseases using cardiac MRI, with explainability analysis.	[25]
Sufian et al.	2024	LIME, SHAP	Cardiovascular MRI	To address algorithmic bias in AI-driven cardiovascular imaging using fairness-aware machine learning methods and explainability techniques such as SHAP and LIME.	[26]
Paciorek et al.	2024	Grad-CAM	Cardiac MRI	To develop and compare deep learning models using DenseNet-161 for automated assessment of cardiac pathologies on T1-mapping and LGE PSIR cardiac MRI sequences.	[21]
Cai et al.	2023	SHAP	Brain MRI and carotid ultrasound	To predict cerebral perfusion status based on internal carotid artery blood flow using machine learning models and explain predictions with SHAP.	[24]
Mouches et al.	2022	Saliency maps (SmoothGrad)	Brain MRI	To predict biological brain age using multimodal MRI data and identify predictive brain and vascular regions.	[23]

### 3.4. Echocardiography and Other Ultrasound Examinations

A substantial proportion of the included studies utilized explainable AI methods in the context of echocardiography or fetal ultrasounds (Table 3). These studies addressed both adult and pediatric cardiovascular diagnostics and spanned a wide range of clinical applications, including valvular heart disease, congenital anomalies, and pulmonary hypertension.

Holste et al. developed a deep learning model using parasternal long axis 2D transthoracic echocardiography videos to detect severe aortic stenosis without relying on Doppler imaging [27]. The model achieved a high diagnostic performance and incorporated Grad-CAM and saliency maps to enhance interpretability, demonstrating its attention to clinically relevant structures, such as the aortic valve.

Focusing on mitral valve morphology, Vafaeezadeh et al. trained a convolutional neural network to classify morphological subtypes based on Carpentier’s functional classification [28]. Grad-CAM was used to visualize the decision-making process, offering transparent insight into how the model distinguished between structural categories.

Another application in structural assessments was presented by Wang et al., who proposed a cardiac segmentation framework that integrated domain knowledge and coordinate attention mechanisms to enhance the U-Net architecture [29]. The model applied to 2D echocardiography data, achieved a strong segmentation performance and explainability through Grad-CAM visualizations.

Ragnarsdottir et al. extended the application of XAI to neonatal cardiology by developing a multi-view video-based deep learning approach for the prediction and grading of pulmonary hypertension in newborns [30]. Grad-CAM was used to confirm that the model focused on key anatomical landmarks across different echocardiographic views.

Three studies applied XAI methods to prenatal cardiac ultrasounds, an area of growing relevance due to the high demand for accurate fetal heart screening. Nurmaini et al. employed a DenseNet architecture to detect congenital heart disease and visualized decision processes via Grad-CAM and guided backpropagation [31]. Similarly, Sakai et al. introduced an innovative graph chart diagram approach based on auto-encoding architectures to enhance examiner performance, providing an intuitive and interpretable representation of fetal cardiac structures [32]. Day et al. (2024) conducted an experimental study to evaluate how AI assistance—including Grad-CAM visualizations—impacted the diagnostic performance of clinicians in identifying atrioventricular septal defects [33]. The inclusion of confidence scores and saliency maps improved collaborative human–AI decision-making.

Next, using apical four-chamber echocardiographic views, Chao et al. developed a deep learning model to differentiate constrictive pericarditis from cardiac amyloidosis [34]. The model achieved an excellent performance (area under the receiver operating characteristic curve [AUC] 0.97 internally, 0.84 externally 0.97 internally, 0.84 externally) and focused on septal motion patterns via Grad-CAM visualizations, supporting its clinical applicability in aiding diagnoses where specialized expertise may be limited.

Lastly, Lee et al. applied CAMs to distinguish incomplete Kawasaki disease from pneumonia in pediatric patients based on short-axis echocardiographic views [35]. Their model demonstrated clinically meaningful visualization outputs, aiding in differential diagnoses in challenging pediatric scenarios.

Taken together, these studies illustrate the growing maturity of XAI applications in echocardiography and ultrasounds, with Grad-CAM emerging as the most commonly used interpretability technique.

**Table 3:** Overview of studies applying explainable AI (XAI) methods to echocardiography and fetal cardiac ultrasound imaging. Abbreviations: XAI: Explainable Artificial Intelligence; Grad-CAM: Gradient-Weighted Class Activation Mapping; CAM: Class Activation Map; 2D echocardiography: Two-dimensional echocardiography; AVSD: Atrioventricular Septal Defect; CNN: convolutional neural network.

Author	Year	XAI Method(s)	Imaging Modality	Aim of the Study	Ref.
Day et al.	2024	Grad-CAM	Fetal Cardiac Ultrasound	To evaluate whether AI advice improves the diagnostic performance of clinicians in detecting fetal atrioventricular septal defect (AVSD) and assess the effect of displaying additional AI model information (confidence and Grad-CAM) on collaborative performance.	[33]
Ragnarsdottir et al.	2024	Grad-CAM	Echocardiography	Automated and explainable prediction of pulmonary hypertension and classification of its severity in newborns.	[30]
Holste et al.	2023	Grad-CAM, Saliency Maps	Echocardiography	To develop and validate an AI model for severe aortic stenosis detection using single-view transthoracic echocardiography without Doppler imaging.	[27]
Chao et al.	2023	Grad-CAM	Echocardiography	Deep learning model (ResNet50) to differentiate constrictive pericarditis from cardiac amyloidosis based on apical four-chamber echocardiographic views.	[34]
Sakai et al.	2022	Graph Chart Diagram	Fetal Cardiac Ultrasound	To improve fetal cardiac ultrasound screening using a novel interpretable deep learning representation to enhance examiner performance.	[32]
Wang et al.	2022	Grad-CAM	2D Echocardiography	To develop a CNN-based cardiac segmentation method incorporating coordinate attention and domain knowledge to improve segmentation accuracy and interpretability.	[29]
Vafaeezadeh et al.	2022	Grad-CAM	Transthoracic Echocardiography	To automatically classify mitral valve morphologies using explainable deep learning based on Carpentier’s functional classification.	[28]
Nurmaini et al.	2022	Grad-CAM, Guided Backpropagation	Prenatal Fetal Ultrasound	To improve prenatal screening for congenital heart disease using deep learning and explain classification via visualization methods.	[31]
Lee et al.	2022	CAM	2D Echocardiography	To distinguish incomplete Kawasaki disease from pneumonia in children using echocardiographic imaging and explainable deep learning.	[35]

### 3.5. Chest X-Ray (CXR)

Four studies applied explainable AI methods to CXR imaging for cardiovascular diagnostics (Table 4). Bhave et al. developed a deep learning model to detect structural abnormalities of the left ventricle, including severe hypertrophy and dilation, using standard CXR images [36]. The model incorporated LayerCAM and CAMs to highlight regions contributing to the prediction, such as the heart borders and thoracic structures, enhancing interpretability and clinical trust.

Matsumoto et al. focused on functional cardiac abnormalities and trained a model to detect atrial fibrillation from posterior–anterior chest radiographs [37]. While AF is not directly visible on X-rays, the model achieved a strong predictive performance. Explainability was ensured using Grad-CAM and guided backpropagation to visualize relevant anatomical areas associated with the prediction, supporting clinical plausibility.

Using a large, multi-institutional dataset, Ueda et al. developed and validated a deep learning model to estimate the cardiac function and valvular heart disease from chest radiographs [38]. The model achieved a strong performance and reliably classified parameters, such as the left ventricular ejection fraction and multiple valvular conditions. Saliency mapping revealed that chest radiographs contain subtle yet informative features for cardiac assessments, demonstrating the potential of explainable AI to complement echocardiography, especially where specialist access is limited.

Lastly, in a study by Kusunose et al., a deep learning model based on chest X-rays was applied to predict exercise-induced pulmonary hypertension (EIPH) in patients with scleroderma or mixed connective tissue disease [39]. By using Grad-CAM for model interpretation, the authors demonstrated that the algorithm primarily focused on cardiac structures in patients with EIPH, providing an explainable insight into the model’s decision-making. Their findings underline the potential of XAI approaches to facilitate early, non-invasive screening for pulmonary vascular diseases in at-risk populations.

These studies demonstrate that even low-cost and widely available imaging modalities, like CXR, can benefit from the integration of explainable deep learning models in cardiovascular risk stratification and diagnosis.

**Table 4:** Overview of studies applying explainable AI (XAI) methods to chest radiography (CXR). Abbreviations: XAI: Explainable Artificial Intelligence; CAM: Class Activation Map; Grad-CAM: Gradient-Weighted Class Activation Mapping; saliency maps: visualization technique highlighting important input regions; CXR: chest X-ray; SLVH: Single Left Ventricular Heart; DLV: Double Left Ventricle.

Author	Year	XAI Method(s)	Imaging Modality	Aim of the Study	Ref.
Bhave et al.	2024	Saliency Mapping, CAM	CXR	To develop and evaluate a deep learning model to detect structural heart abnormalities (SLVH and DLV) from chest X-rays.	[36]
Ueda et al.	2023	Grad-CAM	CXR	Simultaneous classification of cardiac function and valvular diseases from chest X-rays.	[38]
Matsumoto et al.	2022	Grad-CAM, Saliency Maps	CXR	To detect atrial fibrillation using a deep learning model trained on chest X-rays and to visualize the regions of interest using saliency maps.	[37]
Kusunose et al.	2022	Grad-CAM	CXR	To predict exercise-induced pulmonary hypertension in patients with scleroderma using DL on CXR.	[39]

## 4. Discussion

The growing body of literature on XAI in cardiovascular imaging reflects a concerted effort to bridge the “black box” problem of complex deep learning models and the interpretative needs of clinicians. Across the 28 studies included in this review—spanning cardiac CT, MRI, echocardiography, and CXR applications—XAI techniques were employed to render AI decision-making more transparent. In general, these studies demonstrate that contemporary XAI methods can provide clinically plausible explanations for model predictions, thereby addressing the critical question of whether clinicians can trust and understand AI outputs. Indeed, most works used post hoc interpretability tools to highlight image regions or features deemed influential by the model. The prevalence of such approaches is unsurprising—they offer intuitive, image-overlay explanations that align with radiologists’ visual reasoning and are readily supported by open-source tools, factors which have likely spurred their widespread adoption [8]. Notably, simpler inherently interpretable (“white-box”) models were not applied in these studies, which is consistent with observations by Linardatos et al. that little progress has been made in deploying white-box models for complex tasks due to performance trade-offs [5].

Collectively, the included studies provide encouraging evidence that XAI can identify imaging features congruent with cardiovascular pathology, thus adding a layer of confidence to automated interpretations. In cardiac CT and MRI applications, for example, several studies reported that XAI heatmaps concentrated on known anatomical substrates of disease. Such concordance suggests that the AI models are “looking” at the right areas, which is reassuring for clinical use. In echocardiography, deep learning models explained via saliency maps similarly focused on clinically relevant structures—one study’s model for hypertrophic cardiomyopathy localized its attention to the interventricular septum (the hallmark of the disease), while another model identifying valvular disease emphasized abnormal valve contours on the ultrasound. For chest X-rays, XAI techniques helped demystify models that predict cardiac conditions from planar radiographs: for instance, heatmaps for an AI detecting cardiomegaly overlapped the enlarged cardiac silhouette, and a model predicting heart failure from CXR images highlighted features such as pulmonary edema and the cephalization of vessels.

While these results are promising, key limitations and challenges of current XAI approaches are present in cardiovascular imaging. A first notable concern is the heavy reliance on saliency map techniques, which, despite their popularity, come with well-documented drawbacks. Saliency-based explanations tend to be diffuse and qualitative; they highlight broad regions of an image but often lack specificity or a clear delineation of exact features driving a prediction [2]. In complex cardiac images, pathology can be subtle, and a colored heatmap may simply cover an entire ventricle or vessel segment, requiring the clinician to infer the precise cue the model used. Moreover, saliency methods can be sensitive to minor input or parameter changes, potentially reducing the reproducibility of the explanation. As Borys et al. emphasize, most medical imaging XAI to date has centered on such visual explanations, and there is a need to expand toward more diverse forms of explanation that might provide deeper insight or better handle abstract features [3]. For example, case-based reasoning (retrieving and displaying similar past patients’ images) or textual explanations (describing the model’s logic in words) were largely absent from the included studies but could enrich the interpretability beyond heatmaps. An interaction-based XAI approach by Lo and Yin exemplifies how a user-guided exploration of model explanations can support clinical interpretation. Although developed for pneumonia detection in chest X-rays, their concept offers valuable impulses for more interactive and user-centered XAI designs in cardiovascular imaging beyond static saliency maps [40].

The general XAI literature underscores why this is the case: building inherently interpretable yet accurate models for high-dimensional image data is exceedingly difficult, so the community has gravitated toward complex models plus XAI as a compromise [5]. Still, the trade-off between model complexity and interpretability is an ever-present challenge.

Another significant limitation is the lack of a thorough evaluation of the XAI methods themselves within these studies. It is encouraging that most papers qualitatively illustrate their explanations, but far fewer provide quantitative or human-subject evaluations of whether the explanations are valid and useful. In fact, a recent review found that 43% of cardiac AI studies using XAI did not formally assess the quality of the explanations at all [2]. Only a minority engaged domain experts to judge the explanations or employed any sort of ground-truth for explanations (e.g., comparing saliency maps to known lesion locations or to expert annotations). Without measuring XAI’s impact on clinician decision-making or diagnostic accuracy, we cannot be sure that these methods truly enhance clinical practice. Furthermore, the current lack of standardized metrics for explainability makes it difficult to compare methods across studies [2]. 

Despite ongoing challenges, XAI holds significant potential for clinical practice. In cardiovascular imaging, XAI can improve diagnostic confidence and clinician acceptance by making AI decisions more transparent. When an AI system not only detects a subtle finding on cardiac MRI but also highlights the specific region or pattern that informed its decision, physicians are more likely to trust and act upon its output.

Ethical and regulatory considerations also strongly motivate the adoption of XAI. The European Union, with its proposed AI Act, classifies medical AI systems as high-risk and demands a degree of explainability to ensure transparency and reliability. Similarly, the FDA’s evolving approval processes increasingly emphasize understanding AI behavior and failure modes [41,42]. From an ethical perspective, patients have a right to be informed about how AI influences decisions affecting their care. Even when deep models are difficult to fully explain, XAI can support meaningful communication—for example, stating that “the AI noticed a shadow associated with fluid buildup” rather than merely reporting an opaque output [8]. Explainability also contributes to accountability: if algorithms assist in diagnosis, clinicians and institutions must be able to justify AI-influenced decisions. However, superficial explanations risk “explanation washing,” where merely providing an explanation is falsely equated with model trustworthiness. Researchers must therefore ensure that explanations are credible and avoid misleading artifacts. Moreover, user-friendly explanations are not necessarily correct, as highlighted in the general XAI literature [2]. Clinicians must be educated to use XAI outputs as decision aids—not replacements—for clinical judgment.

Future research should focus on methodological standardization for reporting and evaluating XAI. While recent frameworks such as expert-grounded and proxy-grounded evaluations provide a starting point, consensus on effective evaluation metrics is still needed. Shared datasets with annotated explanation masks could enable benchmarking across different algorithms. Furthermore, research should move beyond saliency maps: alternative approaches like case-based reasoning or textual justifications can offer complementary insights, particularly for multimodal datasets combining imaging and clinical data.

Human factors research is also critical to understand how clinicians interact with explanations: for example, whether heatmaps improve diagnostic speed or accuracy. Clinical trials comparing AI systems with and without explainability features could quantitatively assess impacts on diagnostic performance, user trust, and patient outcomes. Validation in real-world environments is essential, as models may behave differently across institutions due to dataset shifts. Continuous monitoring, including periodic audits of explanations, could help maintain reliability over time.

Finally, interdisciplinary collaboration between data scientists, clinicians, and patients will be vital to guide the development of clinically useful XAI tools. No single method will suit all use cases; different explanation types may be needed depending on whether the focus is on subtle imaging abnormalities or a broader patient risk stratification. Engaging end-users through co-design processes can ensure that explanations address relevant clinical questions. Incorporating clinical knowledge into explanation outputs—for instance, relating highlighted areas to anatomical atlases—could further enhance utility.

In summary, XAI is advancing from a conceptual promise toward a practical necessity in cardiovascular imaging. Robust, validated explainability methods can support the trust, ethical standards, and clinical integration of AI in medicine, ensuring that AI systems contribute meaningfully to improved patient care.

## 5. Conclusions and Perspective

This systematic review demonstrates that XAI has been applied across cardiovascular imaging modalities, providing valuable insights into model decision-making through techniques such as Grad-CAM and SHAPs. While these methods often highlight clinically plausible regions and features, their evaluation remains largely qualitative and inconsistent. Saliency-based techniques, although intuitive, exhibit known technical limitations, and the robustness and fidelity of explanations are not yet consistently ensured. The gap between experimental XAI applications and prospective clinical validation remains significant, with few studies assessing the real-world impact of explainability on diagnostic decision-making or clinician trust.

Future research should prioritize standardized evaluation frameworks for XAI quality, integrate explainability features into clinical workflows, and conduct rigorous prospective studies to assess their effect on outcomes. Improving the technical precision of explanations, adopting user-centered design principles, and ensuring that explanations align with clinicians’ cognitive processes will be essential steps toward making AI a reliable partner in cardiovascular diagnostics. Only by addressing these challenges can explainable AI fulfill its potential to bridge the gap between high-performing models and clinical applicability, ultimately enhancing transparency, trust, and patient care in cardiovascular imaging.

## Data Availability

No new data were created or analyzed in this study. Data sharing is not applicable to this article.

## Supplementary Materials

The following supporting information can be downloaded at https://www.mdpi.com/article/10.3390/diagnostics15111399/s1: Table S1: Search strategies used for the systematic review across PubMed, Scopus, and Web of Science, specifying search terms, restrictions, and database-specific adaptations.
