# Supplementary material for: Explainable Artificial Intelligence in Radiological Cardiovascular Imaging—A Systematic Review
Source: Diagnostics (Basel). 2025 May 31;15(11):1399. doi: 10.3390/diagnostics15111399 (PMC12155260; doi:10.3390/diagnostics15111399)
Supplement: Supplementary file 1 [file diagnostics-15-01399-s001.zip › diagnostics-3657942-supplementary.pdf]

Supplementary Material:

Table S1: Search strategies used for the systematic review across PubMed, Scopus, and Web of Science, specifying search terms, restrictions, and database-specific adaptations.

Database	Search String	Restrictions
**PubMed**	((cardiac[Title/Abstract] OR heart[Title/Abstract] OR cardiovascular[Title/Abstract] OR vascular[Title/Abstract] OR vessel[Title/Abstract] OR artery[Title/Abstract] OR arteries[Title/Abstract] OR vein[Title/Abstract] OR veins[Title/Abstract] OR aorta[Title/Abstract] OR "coronary artery"[Title/Abstract] OR "carotid artery"[Title/Abstract] OR "peripheral artery"[Title/Abstract] OR "coronary vessels"[Title/Abstract]) AND ("computed tomography"[Title/Abstract] OR CT[Title/Abstract] OR "CT angiography"[Title/Abstract] OR CTA[Title/Abstract] OR "magnetic resonance imaging"[Title/Abstract] OR MRI[Title/Abstract] OR CMR[Title/Abstract] OR ultrasound[Title/Abstract] OR echocardiography[Title/Abstract] OR "echocardiographic imaging"[Title/Abstract] OR "positron emission tomography"[Title/Abstract] OR PET[Title/Abstract]) AND ("artificial intelligence"[Title/Abstract] OR "machine learning"[Title/Abstract] OR "deep learning"[Title/Abstract] OR "neural network"[Title/Abstract] OR "deep neural network"[Title/Abstract] OR DNN[Title/Abstract] OR "convolutional neural network"[Title/Abstract] OR CNN[Title/Abstract]) AND (XAI OR “Explainable machine learning” OR "explainable AI" OR "explanable AI" OR "explainable artificial intelligence" OR SHAP OR "SHAPley Additive exPlanations" OR LIME OR "layer-wise relevance propagation" OR LRP OR "guided backpropagation" OR "saliency maps" OR "Grad-CAM" OR "Grad-CAM++" OR "DeepLIFT" OR DeConvNet OR SmoothGrad)) AND ("2015"[Date - Publication] : "2025"[Date - Publication]) AND (english[Language]) NOT review[Publication Type]	Timeframe: 2015–2025, Language: English, Reviews excluded

**Scopus**	(TITLE-ABS ("explainable AI" OR "explainable artificial intelligence" OR "Grad-CAM" OR shap OR lime OR "saliency map" OR lrp OR "guided backpropagation" OR deeplift OR deconvnet OR smoothgrad)) AND (TITLE-ABS ("cardiac MRI" OR cmr OR "coronary CT" OR ccta OR echocardiography OR "myocardial perfusion imaging")) AND (TITLE-ABS (image OR imaging OR scan OR modality OR segmentation OR dicom OR "medical image" OR "imaging data"))	Timeframe: 2015–2025, Language: English, Reviews excluded
**Web of Science**	(TI=("explainable AI" OR "Grad-CAM" OR SHAP OR LIME OR "saliency map*" OR LRP OR "guided backpropagation" OR DeepLIFT OR DeConvNet OR SmoothGrad) OR AB=("explainable AI" OR "Grad-CAM" OR SHAP OR LIME OR "saliency map*" OR LRP OR "guided backpropagation" OR DeepLIFT OR DeConvNet OR SmoothGrad)) AND (TS=("cardiac MRI" OR CMR OR "coronary CT" OR CCTA OR "echocardiography" OR "heart ultrasound" OR "myocardial perfusion imaging" OR PET)) AND (TS=("deep learning" OR "convolutional neural network" OR CNN OR "artificial intelligence")) AND PY=(2015-2025) AND LA=(English) NOT DT=(Review)	XAI terms in Title or Abstract; Cardiovascular imaging focus; Timeframe: 2015–2025; Language: English; Reviews excluded
